# Cytoprotective Peptides from Blue Mussel Protein Hydrolysates: Identification and Mechanism Investigation in Human Umbilical Vein Endothelial Cells Injury

**DOI:** 10.3390/md19110609

**Published:** 2021-10-27

**Authors:** Indyaswan Tegar Suryaningtyas, Chang-Bum Ahn, Jae-Young Je

**Affiliations:** 1 Department of Food and Life Science, Pukyong National University, Busan 48513, Korea; indyaswantegar@gmail.com; 2 Division of Food and Nutrition, Chonnam National University, Gwangju 61186, Korea; a321@jnu.ac.kr; 3 Department of Marine-Bio Convergence Science, Pukyong National University, Busan 48547, Korea

**Keywords:** bioactive peptide, cytoprotective, oxidative stress, endothelial dysfunction, blue mussel

## Abstract

Cardiovascular disease represents a leading cause of mortality and is often characterized by the emergence of endothelial dysfunction (ED), a physiologic condition that takes place in the early progress of atherosclerosis. In this study, two cytoprotective peptides derived from blue mussel chymotrypsin hydrolysates with the sequence of EPTF and FTVN were purified and identified. Molecular mechanisms underlying the cytoprotective effects against oxidative stress which lead to human umbilical vein endothelial cells (HUVEC) injury were investigated. The results showed that pretreatment of EPTF, FTVN and their combination (1:1) in 0.1 mg/mL significantly reduced HUVEC death due to H_2_O_2_ exposure. The cytoprotective mechanism of these peptides involves an improvement in the cellular antioxidant defense system, as indicated by the suppression of the intracellular ROS generation through upregulation of the cytoprotective enzyme heme oxygenase-1. In addition, H_2_O_2_ exposure triggers HUVEC damage through the apoptosis process, as evidenced by increased cytochrome C release, Bax protein expression, and the elevated amount of activated caspase-3, however in HUVEC pretreated with peptides and their combination, the presence of those apoptotic stimuli was significantly decreased. Each peptide showed similar cytoprotective effect but no synergistic effect. Taken together, these peptides may be especially important in protecting against oxidative stress-mediated ED.

## 1. Introduction

The imbalance between the antioxidant defense mechanism and reactive oxygen species (ROS) generation in a physiological system leads to oxidative stress and associated disease consequences. Regulated ROS generation is critical for the activation of protective signaling pathways, but when in excess amount it induces oxidative stress. Oxidative stress induces depolarization of the mitochondrial membrane. When the mitochondrial membrane potential is reduced, a series of signaling proteins is activated, which leads to the activation of several stress-responsive genes, such as p53, Bax, Bcl-2, and caspase-3 [1]. This results in enhanced reactive oxygen species generation, severe cell damage, and apoptosis-induced cell death [2,3]. These risk factors can induce endothelial dysfunction (ED) through a variety of processes [4,5]. The endothelium, particularly the terminal arteries, is damaged by too much ROS, which disrupts the intracellular reduction-oxidation balance. Hence, ED is considered as an early indicator in the progression of cardiovascular disease (CVD) [6,7]. Since oxidative stress is defined as a possible cause of cardiovascular disease, treatment with antioxidants is a good strategy to prevent CVD-causing endothelial vein damage.

Recently, marine-derived food proteins have attracted much attention because of their wide range of bioactivity. Seafood consumption is thought to lower the risk of various diseases, as peptides derived from marine-food protein have an anti-inflammatory, antihypertensive, antidiabetic, anticancer, antioxidant, and anti-obesity potential [8,9,10,11,12,13,14]. Some identified antioxidant peptides or protein hydrolysates are important subjects of interest, due to their specific properties as therapeutic agents to protect the body from diseases related to oxidative damage [8,15,16]. Blue mussel (*Mytilus edulis*) is one of the prominent protein-rich marine food sources that can be converted into bioactive peptides (BAPs) through enzymatic hydrolysis to optimize their health benefits, such as anti-inflammation, antioxidant, and anti-obesity [17,18,19]. Fermented blue mussel sauce, a popular Asian-style culinary condiment, has also been associated with CVD risk by producing BAPs with antihypertensive activity [20,21]. An effective strategy to prevent CVD is to protect the venous endothelial cells, which are damaged by oxidative stress. In a previous study, α-chymotrypsin-assisted protein hydrolysate of blue mussel showed cytoprotective activity in protecting HUVECs from damage induced by H_2_O_2_-mediated oxidative stress [1]. However, there is insufficient information in previous reports about specific BAPs that play an important role in endothelial cell protection and certain mechanisms associated with ROS-mediated CVD that need to be understood. The purification of peptides is one of the procedures that needs to be carried out, in order to further expand the use of this compound as a pharmaceutical raw material or functional food source in the future. Therefore, the purpose of this study was to evaluate the capacity of purified peptides derived from blue mussel to protect HUVEC from oxidative stress caused by H_2_O_2_ exposure, as well as to understand the protective mechanism of these peptides.

## 2. Results

### 2.1. Purification and Identification of Cytoprotective Peptides

**Figure 1:**
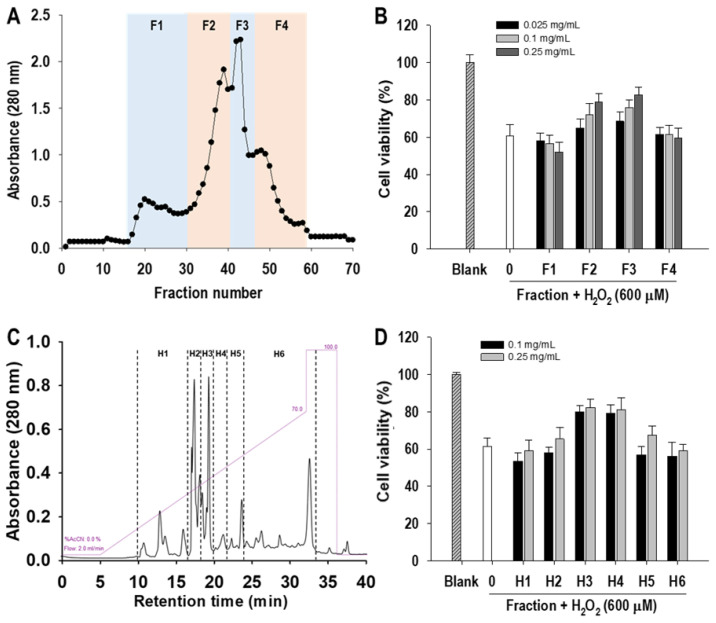
Peptide purification from α-chymotrypsin-assisted blue mussel hydrolysates. (**A**) Gel filtration chromatogram, (**B**) cytoprotective activity of gel filtration fraction, (**C**) HPLC chromatogram, and (**D**) cytoprotective activity of HPLC fraction. Detailed separation conditions are described in Section 2.1. Cells were treated with fraction for 2 h followed by the addition of 600 μM H_2_O_2_ and further incubation for 24 h.

**Figure 2:**
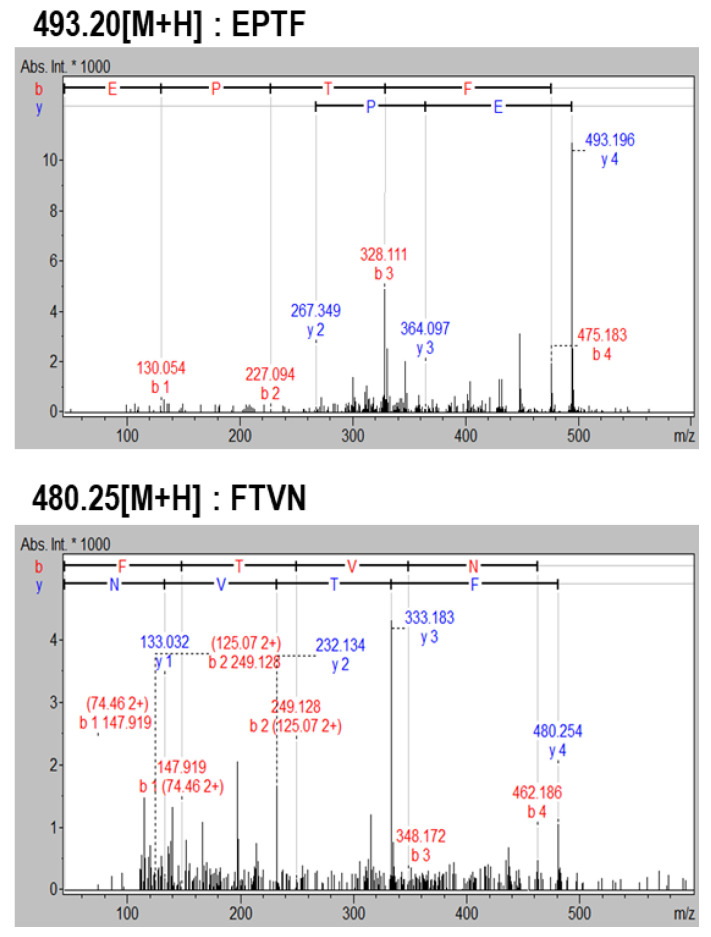
Identification of cytoprotective peptides from α-chymotrypsin-assisted protein hydrolysates of blue mussel by LC-MS/MS.

Cytoprotective peptides were purified from α-chymotrypsin-assisted protein hydrolysates of blue mussel by a cytoprotective activity-guided purification process. First, separation was performed by Sephadex G-25 gel filtration and four fractions (F1~4) were collected. After evaluating cytoprotective activity, the F3 fraction that showed the highest protective effect on HUVEC against H_2_O_2_-induced oxidative cell damage was selected and further purified by HPLC equipped with a C18 column (Figure 1). Six fractions were obtained by HPLC separation. After determination of cytoprotective activity, fractions H3 and H4 showed similar HUVEC protective activity and were analyzed by LC-MS/MS to identify the peptide sequence. Finally, two peptides of EPTF (calculated MW, 493 Da) and FTVN (calculated MW, 480 Da) were identified in H4 fraction (Figure 2). No peptides were identified in the H3 fraction. To evaluate more about their potential to protect HUVECs, these two peptides were chemically synthesized to further investigate their cytoprotective activity and underlying mechanism.

### 2.2. Cytoprotective Activity in H_2_O_2_-Mediated HUVECs Injury

**Figure 3:**
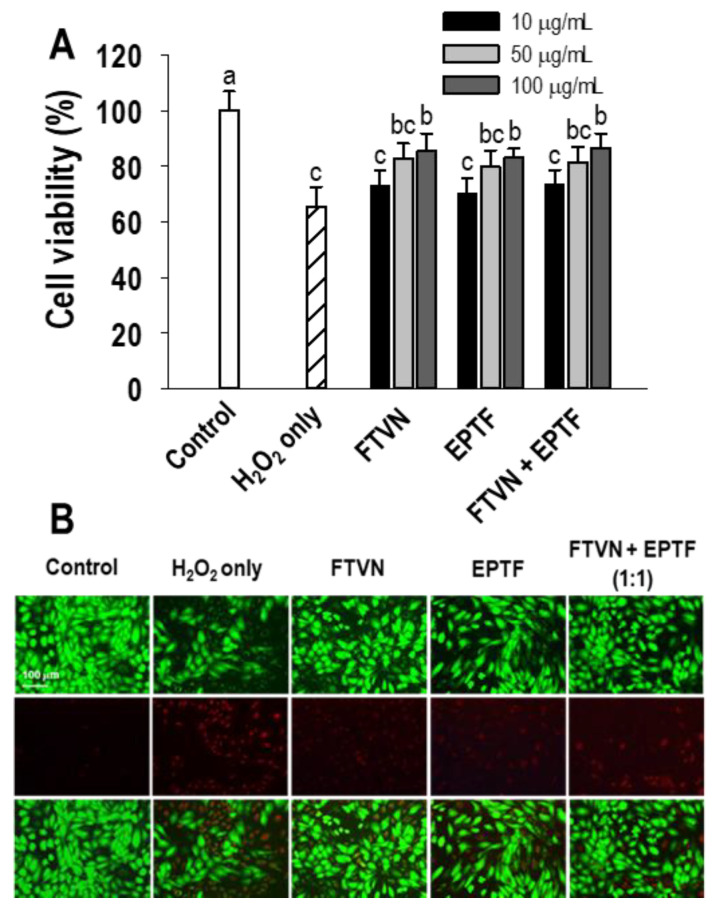
(**A**) The cell viability of HUVECs after BAPs pretreatment at different concentrations. (**B**) Calcein-AM/PI staining assay on EPTF, FTVN, and their combination pretreatment at 0.1 mg/mL. HUVECs were incubated with samples for 2 h before being challenged with 600 μM H_2_O_2_ for 24 h. The data are provided as means ± SD (*n* = 4). ^a–c^ Different letters show significance difference at *p* < 0.05.

Evaluation of cytoprotective activity was carried out on the identified peptides FTVN and EPTF, as well as their combination in the same proportion, to see if there was any synergy effect between the two peptides. Cell viability was evaluated using the MTT assay after cultured HUVECs were treated with sample peptides and subsequently challenged with 600 µM of H_2_O_2_, a concentration which was determined to significantly decrease cell viability in a previous report [22]. Compared with untreated cells that were not exposed to peptides or H_2_O_2_ (control), the addition of H_2_O_2_ significantly decreased the cell viability of HUVEC by 65.43%. Meanwhile, HUVECs pretreated with 100 μg/mL peptide samples showed remarkably increased cell viability of 85.46%, 83.11%, and 86.58% for FTVN, EPTF, and their combination (1:1), respectively. The results indicate the cytoprotective effect of FTVN, EPTF, and their combination. On the other hand, no significant difference was shown in improving HUVECs cell viability between the peptide samples or the combination of FTVN and EPTF. However, the concentration of the samples had a significant effect on cell viability (Figure 3A). Similar results were found in fluorescence microscopy with Calcein-AM/PI double staining analysis, where H_2_O_2_ treatment significantly reduced the green fluorescence of live cells, but pretreatment with FTVN, EPTF, and their combination reversed this effect. Moreover, in H_2_O_2_ treatment, more cells are stained with PI, while the peptides and their combination reduce the cells stained with PI, indicating a cytoprotective effect (Figure 3B).

### 2.3. Inhibition of Intracellular ROS Generation

**Figure 4:**
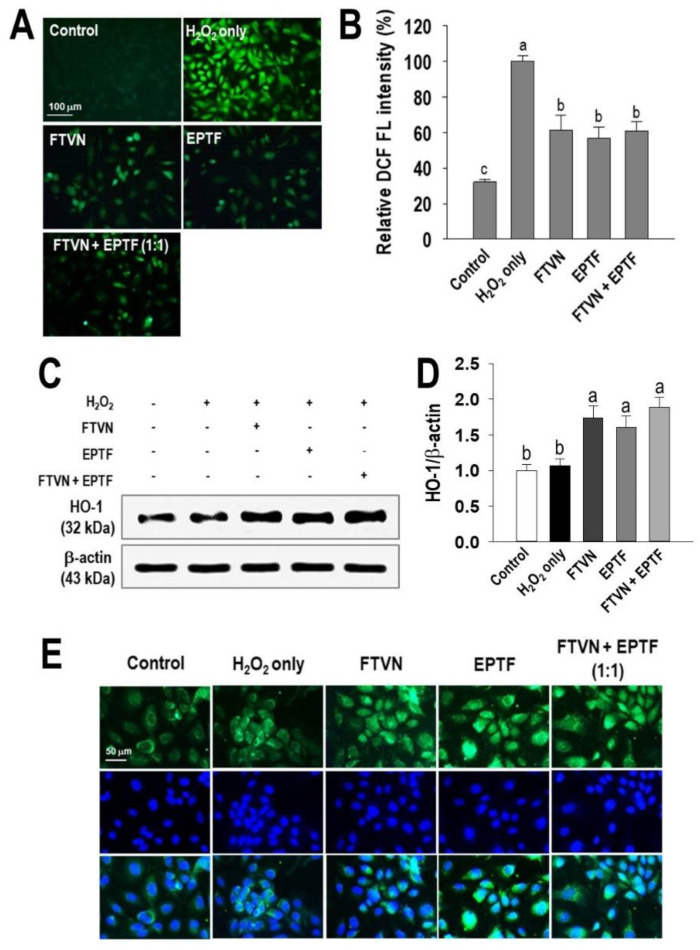
ROS generation in cells and the defense mechanism against it. (**A**) Visualization and (**B**) fluorescence intensity quantification on ROS existence. (**C**) Western blot analysis and (**D**) HO-1 expression value. (**E**) Nrf2 nuclear accumulation immunofluorescence stained HUVECs were incubated with 0.1 mg/mL of peptides for 2 h before being challenged with 600 μM H_2_O_2_ for 24 h (ROS generation and HO-1 analysis) or within 2 h for Nrf2 analysis. The data are provided as means ± SD (*n* = 4). ^a–c^ (ROS generation) and ± SD (*n* = 3). ^a–c^ (HO-1 and Nrf2 analysis). Different letters show significance difference at *p* < 0.05.

It is hypothesized that ROS in cells is one of the causes of H_2_O_2_-induced HUVEC damage. Therefore, we also investigated the presence of ROS in the cells. The DCFH-DA fluorescent probe revealed the amount of intracellular ROS was significantly higher in HUVECs exposed to H_2_O_2_ alone compared to the control group. This is indicated by the visibility of the DCF fluorescence signal. The DCF fluorescence signal was decreased in HUVECs with pretreatment of FTVN and EPTF or their combination in H_2_O_2_-induced HUVEC injury (Figure 4A). Quantitatively, ROS generation in the cells significantly increased as a result of H_2_O_2_ exposure to HUVECs, whereas pretreatment of FTVN, EPTF, and the combination of both significantly reduced the intracellular ROS generation by approximately 40% (Figure 4B).

### 2.4. HO-1/Nrf2 Pathway Activation by Cytoprotective Peptides

HO-1 is one of the phase II detoxifying enzymes. In a ROS detoxifying mechanism, this enzyme must be induced by activation of a transcription factor such as Nrf2. This event is regarded as one of the most important cellular defense mechanisms [23]. As shown in Figure 4C,D, HO-1 expression slightly increased in the presence of H_2_O_2_ treatment alone, indicating spontaneous reaction for cytoprotection, and this finding is similar to a previous report [1]. However, the amount of HO-1 induction in the presence of H_2_O_2_ treatment alone is not enough to protect HUVECs, but we demonstrated that peptide treatment can increase the induction of HO-1, indicating the cytoprotective effect against exogenous stimuli. The induction of HO-1 is further confirmed by evidence of the translocation of Nrf2 into the nucleus, which regulates the expression of HO-1. As shown in Figure 4E, Nrf2 was detectable in the cytoplasm in the non-treated and H_2_O_2_ treated cells, whereas Nrf2 was slightly detectable in the nucleus in the H_2_O_2_ treated cells. Compared with the cells treated with H_2_O_2_ alone, treatment with FTVN and EPTF or their combination resulted in upregulation of Nrf2 expression in both the cytoplasm and nucleus of HUVECs, indicating activation of Nrf2 followed by induction of HO-1.

### 2.5. Anti-Apoptotic Activity against H_2_O_2_-Induced HUVECs Damage

A high dose exposure of powerful oxidants such H_2_O_2_ causes severe damage to macromolecules, which leads to cell death through apoptosis and/or necrosis mechanisms [24]. The cytoprotective activity of EPTF, FTVN, as well as their combination was evaluated by measuring the anti-apoptotic activity. Annexin V-FITC/PI and Hoechst 33342 staining was performed after the peptide treatments.

**Figure 5:**
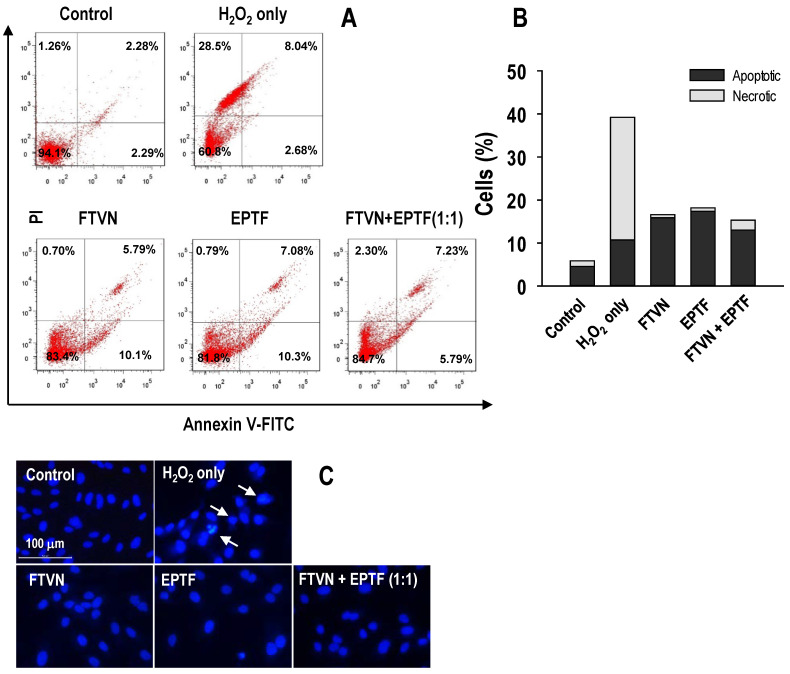
The effect of EPTF, FTVN, and combination in inhibiting HUVEC apoptosis. (**A**) Quadrant dot analysis showing live–dead cells, and (**B**) apoptotic and necrotic ratio using flow cytometer analysis using Annexin V-FITC/PI staining assay. (**C**) Morphological changes under fluorescence microscope with Hoechst 33342 staining assay (white arrows showed apoptosis occurrence). HUVECs were pretreated with 0.1 mg/mL of peptides for 2 h before being challenged with 600 μM H_2_O_2_ for 24 h. All treatment was carried out in triplicate.

**Figure 6:**
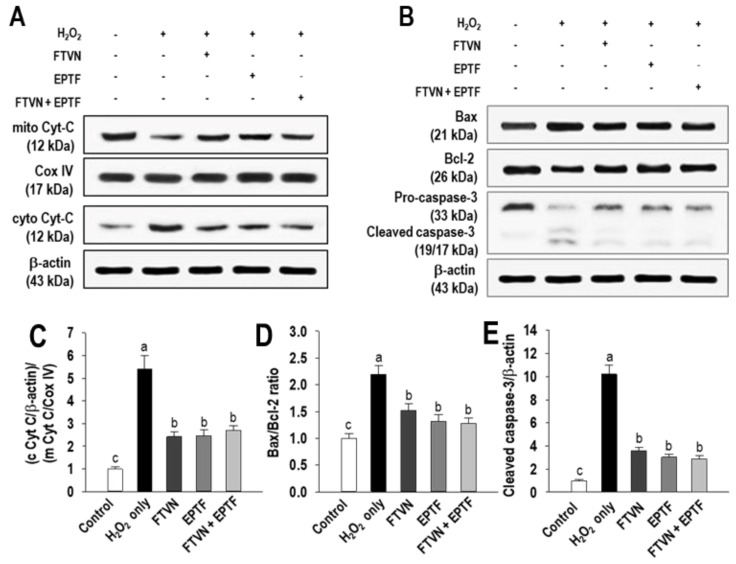
Western blot analysis of (**A**) released mitochondrial cytochrome C (Cyt-C) found in cytosol, and (**B**) Bax, Bcl-2, and activated caspase-3 (procaspase-3/cleaved caspase-3) expression. As protein loading control, Cox IV and β-actin were used. Expression level of (**C**) released Cyt C within mitochondria and cytoplasm, (**D**) ratio of Bax/Bcl-2 expression, and (**E**) cleaved caspase-3/β-actin for caspase-3 activation. HUVECs were incubated with 0.1 mg/mL of peptides for 2 h before being challenged with 600 μM H_2_O_2_ for 24 h. All treatment was carried out in triplicate. The data are provided as means ± SD (*n* = 3). Different letters show significance difference at *p* < 0.05.

The results revealed that 94.1% of HUVECs were located in the lower left quadrant in the non-treated cells (control), which decreased to 60.8% in the H_2_O_2_ group. There was a high percentage of necrotic cell (28.5%) compared to apoptotic cell (10.72%) in the presence of H_2_O_2_ treatment alone. Pretreatment of HUVECs with EPTF, FTVN, and their combination significantly reduced the total percentage of dead cells compared to the H_2_O_2_ treatment group (Figure 5A). This indicates that our model treatment leads to apoptosis first, followed by necrosis. However, peptide treatment decreased predominantly the necrosis rate (Figure 5B). Figure 6 confirmed that peptide treatment modulated the protein expression related to apoptosis, indicating that cell survival by peptide treatment is attributed to downregulation of apoptotic protein expression.

Observation of nuclear morphology with a fluorescence microscope shows that H_2_O_2_ treatment leads to morphological changes, especially nuclear shrinkage, segregation, and chromatin condensation (Figure 5C). HUVECs in the peptides-pretreated group were comparable to those in the non-treated cells (control), suggesting that EPTF, FTVN, and their combination protect the HUVECs from apoptotic cell death.

It is known that disruption of mitochondrial membrane integrity by H_2_O_2_ insult leads to the release of cytochrome C (Cyt C), which in turn causes apoptosis in cells [25]. Western blot analysis was performed to investigate the effect of EPTF, FTVN, and their combination on the released of Cyt C into the cytoplasm. As shown in Figure 6A, mitochondrial Cyt C was strongly detected in the non-treated cells, while cytosolic Cyt C was weakly detected. On the other hand, H_2_O_2_ treatment resulted in cytoplasmic release of Cyt C from mitochondria into the cytoplasm, and Cyt C was strongly detected in the cytoplasm, indicating activation of apoptosis mediated by the intrinsic pathway. However, the release of the Cyt C from mitochondria in the cytoplasm by H_2_O_2_ treatment was significantly reduced after pretreatment with EPTF, FTVN, and their combination (Figure 6A,C), indicating suppression of the intrinsic pathway by H_2_O_2_ exposure.

The expression ratio of Bcl-2 and Bax plays a crucial role in the apoptosis process by regulating mitochondrial membrane permeability, which is associated with the disruption of mitochondrial membrane integrity [26]. In this study, H_2_O_2_ exposure resulted in an increase in Bax expression but a decrease in Bcl-2 expression (Figure 6B). In the cells pretreated with EPTF, FTVN, and their combination, the Bax and Bcl-2 expression was reversed and the Bax/Bcl-2 ratio was also significantly reduced compared to the cells with H_2_O_2_ treatment (Figure 6B,D). Finally, this study examined the activation of caspase-3, which is known to be the execution caspase in apoptosis. High expression of procaspase-3 was detected in the untreated cells, whereas the cleaved caspase-3, the active form of caspase-3, was negligible (Figure 6B,E). On the other hand, procaspase-3 was converted to cleaved caspase-3 in the presence of H_2_O_2_ treatment, but this process was abolished by pretreatment with EPTF, FTVN, and their combination, suggesting that the anti-apoptotic effect of the peptides came from suppression of the caspase-3 pathway.

## 3. Discussion

Recent studies have shown that BAPs derived from marine dietary proteins by enzymatic hydrolysis have versatile health benefits, as they have antioxidant, antihypertensive, and antidiabetic effects. To date, many BAPs have been isolated and identified from marine dietary protein hydrolysates [27]. In addition, previous studies have identified specific BAPs in blue mussel protein hydrolysates that have been attributed antioxidant, antithrombotic, antihypertensive, osteogenic, and anti-osteoporotic effects [19,20,28,29,30,31]. Blue mussel protein hydrolysate produced by α-chymotrypsin has been previously identified as a potential cytoprotective agent [1]. However, there is limited information on specific BAPs with cytoprotective effects of blue mussel protein hydrolysates in alleviating HUVEC damage caused by oxidative stress. In this study, two cytoprotective peptides were isolated and identified as EPTF and FTVN, and their molecular mechanism underlying the cytoprotective activity was investigated.

The idea of using antioxidants with cryoprotective affects to treat CVD is based on the evidence that the excess amount of ROS generates oxidative stress, which then leads to endothelial cell damage and induces apoptosis [31]. Endothelial dysfunction (ED) a physiological condition that occurs in the early development of atherosclerosis, is often characterized by cell death, including the mechanism of apoptosis [32]. H_2_O_2_ is one of the most understood ROS, serving as a second messenger in a variety of critical cellular signaling pathways, but when it presents in high concentrations it has toxic consequences that can lead to cellular dysfunction or even cell death. Therefore, to evaluate the cytoprotective effects of EPTF and FTVN, an H_2_O_2_-mediated HUVEC injury model was used. Since these two peptides were identified in the same fraction (H4), we investigated the cytoprotective effect of each peptide as well as their combination (synergic effect). It was found that pretreatment with EPTF, FTVN and both combinations reversed the cell death induced by H_2_O_2_ treatment. Moreover, increased ROS generation by H_2_O_2_ treatment was remarkedly quenched by pretreatment of EPTF, FTVN, and their combination. There was no significant difference in the cytoprotective effect between the peptides and their combination. Each peptide had a potent cytoprotective effect on its own. This indicates that the contribution of each peptide to cytoprotective is similar.

To uncover the mechanism underlying the cytoprotective activity, the effect of EPTF, FTVN, and their combination in the cellular antioxidant defense system was investigated. Nuclear factor erythroid 2-related factor 2 (Nrf2) is a regulator of species lifespan that regulates the expression of genes coding for detoxifying proteins, antioxidants, and anti-inflammatories [33]. In this study, H_2_O_2_ plays a role as an exogenous stimulus that induces the oxidative damage and triggers the activation of Nrf2. In this condition, the Nrf2-Keap1-Cullin3 complex was disrupted, which then allowed the translocation of Nrf2 from cytoplasm to nucleus [34]. From Figure 4E, we can see that Nrf2 was accumulated in nucleus as an indication of cytoprotective activity. One of the genes regulated through Nrf2 is HO-1, which is recognized as a cytoprotective enzyme through catalyzing heme protein into ferritin, biliverdin, and carbon monoxide [35]. In our study, H_2_O_2_ stimulated the production of HO-1 but only with insignificant concentration; this result is similar to another study using H_2_O_2_ as the stress model [36,37]. This might be due to the very high concentrations of exogenous stimuli that exceed the cell capacity to perform cytoprotective mechanisms, which also results in cellular damage. To eliminate damaged cells, the apoptosis process occurs. We demonstrated that pretreatment of EPTF, FTVN, and their combination upregulated HO-1 expression through Nrf2 activation in H_2_O_2_-mediated HUVEC injury.

Induction of HO-1 by peptide treatment may have influenced cell survival and the decrease in intracellular ROS generation. Mitochondria is a natural source of cellular ROS; their membranes contain certain key compounds involved in antioxidant responses and in stimulating apoptotic pathways [38]. When oxidative stress surpasses a cell’s ability to balance it, mitochondrial dysfunction occurs, which then leads mitochondria to generate more ROS. Furthermore, oxidative stress promotes nuclear damage and triggers the cascade of apoptotic cell death. Apoptosis is a programmed cell death that is governed by two major pathways including the intrinsic mitochondria pathway and the extrinsic death receptor pathway [39]. Since H_2_O_2_ is known to induce apoptosis through the intrinsic mitochondria pathway, in this study the role of EPTF, FTVN, and their combination was investigated in the intrinsic mitochondria pathway [40]. A key event of this pathway is mitochondria Cyt C released into the cytoplasm through the relative ratio of Bax and Bcl-2 proteins expression, i.e., a high Bax/Bcl-2 ratio increased cell death probability through an intrinsic mitochondria pathway-mediated apoptosis [41]. The released Cyt C then binds to apoptotic protease-activating factor-1 and forms an apoptosome with procaspase-9. This activates caspase-3, an important trigger of apoptosis. In this study, H_2_O_2_ exposure increased Bax expression while decreasing Bcl-2 expression in HUVEC and showed a high ratio of Bax/Bcl-2, indicating the increase of mitochondrial membrane permeability. However, its value was decreased in HUVEC that received pretreatment with EPTF, FTVN, and their combination. In addition, our data clearly showed H_2_O_2_-mediated mitochondria dysfunction, which is proved by the accumulation of Cyt C in the cytoplasm, while pretreatment of EPTF, FTVN, and their combination inhibited this event in the cytoplasm.

Finally, H_2_O_2_-mediated activation of caspase-3 in HUVEC was inhibited by cascade activation via alteration of the Bax-Bcl-2 ratio and release of Cyt C by pretreatment of EPTF, FTVN, and their combination. This suggests that the intrinsic mitochondrial pathway is involved in the cytoprotective effect induced by EPTF, FTVN and their combination in H_2_O_2_-mediated HUVEC damage.

Similar results of BAPs from various food proteins, including edible seahorse, rice bran, and *Mytilus coruscus*, have been reported in H_2_O_2_-mediated HUVEC injury where these BAPs showed the cytoprotective effect through modulation of the intrinsic mitochondria pathway [24,42,43]. These findings suggest that BAPs may be useful for ameliorating oxidative stress-mediated ED and may be helpful for treating CVD.

## 4. Material and Methods

### 4.1. Materials

Blue mussel (*Mytilus edulis*) was purchased from Yeosu Fisheries Co. (Yeosu, Korea). Calcein AM solution, propidium iodide (PI), 2′7′-dichlorofluorescein diacetate (DCFH-DA), H_2_O_2_, Hoechst 33342, and 3-(4,5-dimethylthiazol-2-yl)-2,5-diphenyltetrazolium bromide (MTT) were purchased from Sigma-Aldrich Chemical Co. (St. Louis, MO, USA). Trypsin-EDTA solution and PBS were purchased from Hyclone (Logan, UT, USA). The HUVEC (PCS-100-010™) are produced by the American Type Culture Collection (Rockville, MD, USA). Endothelial Growth Medium-2 Bullet Kit (EGM™-2) was produced by Lonza, (Walkersville, MD, USA).

### 4.2. Blue Mussel Protein Hydrolysate by α-Chymotrypsin-Assisted Hydrolysis

Blue mussel was rinsed and lyophilized before being hydrolyzed by α-chymotrypsin (pH 8.0 and 37 °C), with 1:100 (enzyme to substrate ratio) and 8 h incubation [1]. Enzyme activity was stopped by 10 min boiling in 100 °C. The supernatants were collected by centrifugation (5000 rpm for 20 min, Labogene 1248R, Seoul, Korea), lyophilized and kept at −20 °C before use.

### 4.3. Purification and Identification of Cytoprotective Peptides

Blue mussel hydrolysates were eluted at 1.0 mL/min rate over Sephadex G-25 column (3.0 × 90 cm), then every 5 min the eluate was collected. Fractions with cytoprotective activity were separated using HPLC equipped with C_18_ column at 2.0 mL/min flow rate (Hypersil Gold, 250 × 10 mm, 5 µm, Thermo Scientific, Pittsburgh, PA, USA). A linear gradient elution was carried out using acetonitrile, as mentioned in a previous publication [24]. Q-TOF LC-MS/MS coupled with an ESI source (maXis-HD™, Bruker Daltonics, Bremen, Germany) was used to perform peptide identification, and subsequently MS/MS spectrometry was used in peptide sequencing (Biotools 3.2, Bruker Daltonics, Bremen, Germany) [18]. The synthesized peptides were ordered from Biostem (Ansan, Korea). HPLC-MS/MS was used to check the purity of the synthesized peptides (over 96% purity).

### 4.4. HUVECs Culture and Treatment

HUVECs were cultured in 37 °C and 5% CO_2_ incubation, using EGM™-2 Medium Kit. The cells were subcultured and harvested using a 0.025% trypsin-EDTA solution. For experimental design, only HUVECs at passages 3–5 were used. They were then seeded in a 96- or 24-well plate, or 60 mm^2^ dishes. Sample peptides were added for the pretreated group following 2 h incubation before being challenged with 600 μM H_2_O_2_ for 24 h. The control group was cells without peptides treatment and H_2_O_2_ exposure.

### 4.5. Cell Viability Assessment

Cytoprotective effect was evaluated by monitoring cell viability using MTT assay to HUVECs in a 96-well plate with 1 × 10^4^ cells/well density. For further confirmation of the cytoprotective effect, live and dead cell assay was also performed. HUVECs were rinsed with warmed PBS in a 24-well plate with 2 × 10^4^ cells/well, and then double stained using calcein-AM and PI 2.5 μM and 5 μM, respectively, following 30 min incubation at 37 °C. Stained cells were distinguished as live and dead cells under a fluorescence microscope (Leica DMI6000 B, Wetzlar, Germany).

### 4.6. Determination of Intracellular ROS

ROS generation in cells was detected in a 96-well black plate for quantification and a 24-well plate for microscopic observation. Pretreated cells were mixed with Hank’s Balanced Salt Solution contained 20 μM DCFH-DA followed by 20 min incubation (37 °C). Fluorescence intensity was measured to determined intracellular ROS level at 485 and 528 nm (excitation and emission) (GENios, TECAN, Männedorf, Switzerland) and visually observed under fluorescence microscope (Leica DMI6000 B, Wetzlar, Germany).

### 4.7. Nrf2 Nuclear Translocation Assessment

To observe Nrf2 nuclear translocation, HUVECs culture was mixed with 3.7% paraformaldehyde in PBS for 15 min, and then permeabilized using 0.1% Triton X-100 dissolve with PBS (10 min), before being blocked in 2% bovine serum albumin with 30 min incubation. Later, anti-Nrf2 antibody with 1:200 dilution was added. After overnight incubation at 4 °C, the secondary antibody (Alexa Fluor^®^ 488, Santa Cruz Biotechnology, Santa Cruz, CA, USA) was added (1:500 dilution with 1h incubation). To counterstain the nuclei, 2 μg/mL Hoechst 33342 was added following 10 min incubation. Visual observation was performed under fluorescence microscope (Leica DMI6000 B, Wetzlar, Germany).

### 4.8. Annexin V-FITC/PI for Apoptotic Cells

Treated cells were washed three times using PBS, harvested, and then resuspended with Annexin V-FITC and PI in binding buffer solution followed by 15 min incubation at room temperature. Apoptotic cells were then analyzed by a flow cytometry (FACSCalibur system, BD Biosciences, San Jose, CA, USA) using an Apoptosis Detection Kit (BD Pharmingen™, San Jose, CA, USA).

### 4.9. Hoechst 33342 Staining Analysis

Treated cells were then washed, harvested, and then fixated using ethanol. After incubation for 20 min, 10 μM Hoechst 33342 staining was added following another 20 min incubation in room temperature. Observation was perform using a fluorescence microscope (Leica DMI6000 B, Wetzlar, Germany).

### 4.10. Western Blot Analysis

Total proteins were extracted with a RIPA buffer (Sigma Chemical Co., St. Louis, MO, USA). The mitochondria and cytosol fractions were isolated using a Mitochondria Isolation Kit (Abcam, Cambridge, MA, USA) following the manufacturer’s procedure. Western blotting was conducted as describe in our previous report [24]. Briefly, 25 μg of total extracted proteins were separated via 10–12% SDS-PAGE prior to nitrocellulose membranes transfer by electroblotting, which was then blocked with 5% skimmed milk for 1 h. Blots were then incubated with specific antibodies HO-1 (1:200), Bax (1:200), Bcl-2 (1:200), cytochrom C (1:200), β-actin (1:500), and Cox IV (1:500) (Santa Cruz Biotechnology, Santa Cruz, CA, USA) and caspase-3 (1:500) (Cell Signaling Technology, Beverly, MA, USA) overnight at 4 °C;. The horseradish peroxidase-conjugated antibodies were regarded as the secondary antibody. The bands were detected by chemiluminescence using the ECL Western blotting assay kit (Life Technologies, Seoul, Korea) and visualized by Davinch-Chemi Imager™ (CAS-400SM, Core Bio, Seoul, Korea).

### 4.11. Data Analysis

SigmaPlot^®^ 12.0 (Systat Software Inc., San Jose, CA, USA) software was used to perform statistical analysis of the data. All experiments are expressed as mean ± standard deviation (SD). Student’s *t*-test was performed, and statistical significance was assigned in accordance with *p* < 0.05.

## 5. Conclusions

The findings of this study demonstrated the cytoprotective activity of the BAPs identified as FTVN and EPTF from blue mussel protein and their role in preventing oxidative stress-mediated endothelial dysfunction (ED). Investigations of the cytoprotective mechanism of these two peptides and their combination in H_2_O_2_-mediated HUVEC injury revealed that BAPs alleviated HUVEC injury through enhancement of the antioxidant defense system and anti-necrotic action. As a result, BAPs derived from blue mussel protein might be an alternative approach in preventing CVD through protecting cardiovascular vein endothelial cells. Furthermore, to develop a nutraceutical or pharmaceutical component based on this result, more in vivo research is required.

## Data Availability

The original contribution presented in this study are included in the article, further inquiries can be directed to the corresponding author.

## References

[1] Oh Y., Ahn C.-B., Nam K.-H., Kim Y.-K., Yoon N.Y., Je J.-Y. (2019). Amino acid composition, antioxidant, and cytoprotective effect of blue mussel (Mytilus edulis) hydrolysate through the inhibition of caspase-3 activation in oxidative stress-mediated endothelial cell injury. Mar. Drugs.

[2] Wang J., Toan S., Zhou H. (2020). New insights into the role of mitochondria in cardiac microvascular ischemia/reperfusion injury. Angiogenesis.

[3] Paone S., Baxter A.A., Hulett M.D., Poon I.K. (2019). Endothelial cell apoptosis and the role of endothelial cell-derived extracellular vesicles in the progression of atherosclerosis. Cell. Mol. Life Sci..

[4] Oh Y.S., Jun H.-S. (2018). Effects of glucagon-like peptide-1 on oxidative stress and Nrf2 signaling. Int. J. Mol. Sci..

[5] Xu F., Zhang J., Wang Z., Yao Y., Atungulu G.G., Ju X., Wang L. (2018). Absorption and metabolism of peptide WDHHAPQLR derived from rapeseed protein and inhibition of HUVEC apoptosis under oxidative stress. J. Agric. Food Chem..

[6] Togliatto G., Lombardo G., Brizzi M.F. (2017). The future challenge of reactive oxygen species (ROS) in hypertension: From bench to bed side. Int. J. Mol. Sci..

[7] Wang K., Dong Y., Liu J., Qian L., Wang T., Gao X., Wang K., Zhou L. (2020). Effects of REDOX in regulating and treatment of metabolic and inflammatory cardiovascular diseases. Oxidative Med. Cell. Longev..

[8] Jin J.-E., Ahn C.-B., Je J.-Y. (2018). Purification and characterization of antioxidant peptides from enzymatically hydrolyzed ark shell (Scapharca subcrenata). Process Biochem..

[9] Hyung J.-H., Ahn C.-B., Kim B.I., Kim K., Je J.-Y. (2016). Involvement of Nrf2-mediated heme oxygenase-1 expression in anti-inflammatory action of chitosan oligosaccharides through MAPK activation in murine macrophages. Eur. J. Pharmacol..

[10] Kim Y.-S., Ahn C.-B., Je J.-Y. (2016). Anti-inflammatory action of high molecular weight Mytilus edulis hydrolysates fraction in LPS-induced RAW264. 7 macrophage via NF-κB and MAPK pathways. Food Chem..

[11] Je J.-Y., Park S.Y., Hwang J.-Y., Ahn C.-B. (2015). Amino acid composition and in vitro antioxidant and cytoprotective activity of abalone viscera hydrolysate. J. Funct. Foods.

[12] Ahn C.-B., Cho Y.-S., Je J.-Y. (2015). Purification and anti-inflammatory action of tripeptide from salmon pectoral fin byproduct protein hydrolysate. Food Chem..

[13] Oh Y., Shim K.-B., Ahn C.-B., Kim S.S., Je J.-Y. (2019). Sea squirt (Halocynthia roretzi) hydrolysates induce apoptosis in human colon cancer HT-29 cells through activation of reactive oxygen species. Nutr. Cancer.

[14] Je J.-Y., Qian Z.-J., Lee S.-H., Byun H.-G., Kim S.-K. (2008). Purification and antioxidant properties of bigeye tuna (Thunnus obesus) dark muscle peptide on free radical-mediated oxidative systems. J. Med. Food.

[15] Fernando I.P.S., Park S.Y., Han E.J., Kim H.-S., Kang D.-S., Je J.-Y., Ahn C.-B., Ahn G. (2020). Isolation of an antioxidant peptide from krill protein hydrolysates as a novel agent with potential hepatoprotective effects. J. Funct. Foods.

[16] Kim S.S., Ahn C.-B., Moon S.W., Je J.-Y. (2018). Purification and antioxidant activities of peptides from sea squirt (Halocynthia roretzi) protein hydrolysates using pepsin hydrolysis. Food Biosci..

[17] Park S.Y., Ahn C.B., Je J.Y. (2014). Antioxidant and anti-inflammatory activities of protein hydrolysates from Mytilus edulis and ultrafiltration membrane fractions. J. Food Biochem..

[18] Oh Y., Ahn C.-B., Je J.-Y. (2020). Low molecular weight blue mussel hydrolysates inhibit adipogenesis in mouse mesenchymal stem cells through upregulating HO-1/Nrf2 pathway. Food Res. Int..

[19] Park S.Y., Kim Y.-S., Ahn C.-B., Je J.-Y. (2016). Partial purification and identification of three antioxidant peptides with hepatoprotective effects from blue mussel (Mytilus edulis) hydrolysate by peptic hydrolysis. J. Funct. Foods.

[20] Je J.-Y., Park P.-J., Byun H.-G., Jung W.-K., Kim S.-K. (2005). Angiotensin I converting enzyme (ACE) inhibitory peptide derived from the sauce of fermented blue mussel, Mytilus edulis. Bioresour. Technol..

[21] Magtaan J.K., Fitzpatrick B., Murphy R. (2021). Elucidating the Biological Activity of Fish-Derived Collagen and Gelatine Hydrolysates using Animal Cell Culture-A Review. Curr. Pharm. Des..

[22] Oh Y., Ahn C.-B., Yoon N.Y., Nam K.H., Kim Y.-K., Je J.-Y. (2018). Protective effect of enzymatic hydrolysates from seahorse (Hippocampus abdominalis) against H_2_O_2_-mediated human umbilical vein endothelial cell injury. Biomed. Pharmacother..

[23] Keum Y.-S. (2012). Regulation of Nrf2-mediated phase II detoxification and anti-oxidant genes. Biomol. Ther..

[24] Oh Y., Ahn C.-B., Je J.-Y. (2021). Cytoprotective Role of Edible Seahorse (Hippocampus abdominalis)-Derived Peptides in H_2_O_2_-Induced Oxidative Stress in Human Umbilical Vein Endothelial Cells. Mar. Drugs.

[25] Garrido C., Galluzzi L., Brunet M., Puig P., Didelot C., Kroemer G. (2006). Mechanisms of cytochrome c release from mitochondria. Cell Death Differ..

[26] Chen Q., Liu X.-F., Zheng P.-S. (2014). Grape seed proanthocyanidins (GSPs) inhibit the growth of cervical cancer by inducing apoptosis mediated by the mitochondrial pathway. PLoS ONE.

[27] Admassu H., Gasmalla M.A.A., Yang R., Zhao W. (2018). Bioactive peptides derived from seaweed protein and their health benefits: Antihypertensive, antioxidant, and antidiabetic properties. J. Food Sci..

[28] Qiao M., Tu M., Wang Z., Mao F., Chen H., Qin L., Du M. (2018). Identification and antithrombotic activity of peptides from blue mussel (Mytilus edulis) protein. Int. J. Mol. Sci..

[29] Oh Y., Ahn C.-B., Je J.-Y. (2020). Blue Mussel-Derived Peptides PIISVYWK and FSVVPSPK Trigger Wnt/β-Catenin Signaling-Mediated Osteogenesis in Human Bone Marrow Mesenchymal Stem Cells. Mar. Drugs.

[30] Oh Y., Ahn C.-B., Cho W.H., Yoon N.Y., Je J.-Y. (2020). Anti-Osteoporotic Effects of Antioxidant Peptides PIISVYWK and FSVVPSPK from Mytilus edulis on Ovariectomized Mice. Antioxidants.

[31] Wang B., Li L., Chi C.-F., Ma J.-H., Luo H.-Y., Xu Y.-f. (2013). Purification and characterisation of a novel antioxidant peptide derived from blue mussel (Mytilus edulis) protein hydrolysate. Food Chem..

[32] Shah D., Das P., Alam M.A., Mahajan N., Romero F., Shahid M., Singh H., Bhandari V. (2019). MicroRNA-34a promotes endothelial dysfunction and mitochondrial-mediated apoptosis in murine models of acute lung injury. Am. J. Respir. Cell Mol. Biol..

[33] Loboda A., Damulewicz M., Pyza E., Jozkowicz A., Dulak J. (2016). Role of Nrf2/HO-1 system in development, oxidative stress response and diseases: An evolutionarily conserved mechanism. Cell. Mol. Life Sci..

[34] da Costa R.M., Rodrigues D., Pereira C.A., Silva J.F., Alves J.V., Lobato N.S., Tostes R.C. (2019). Nrf2 as a potential mediator of cardiovascular risk in metabolic diseases. Front. Pharmacol..

[35] Ryter S.W., Choi A.M. (2016). Targeting heme oxygenase-1 and carbon monoxide for therapeutic modulation of inflammation. Transl. Res..

[36] Choi Y.H. (2019). Activation of the Nrf2/HO-1 signaling pathway contributes to the protective effects of coptisine against oxidative stress-induced DNA damage and apoptosis in HaCaT keratinocytes. Gen. Physiol. Biophys..

[37] Yuan J., Lu Y., Wang H., Feng Y., Jiang S., Gao X.-H., Qi R., Wu Y., Chen H.-D. (2020). Paeoniflorin resists H_2_O_2_-induced oxidative stress in melanocytes by JNK/Nrf2/HO-1 pathway. Front. Pharmacol..

[38] Calabrese G., Peker E., Amponsah P.S., Hoehne M.N., Riemer T., Mai M., Bienert G.P., Deponte M., Morgan B., Riemer J. (2019). Hyperoxidation of mitochondrial peroxiredoxin limits H_2_O_2_-induced cell death in yeast. Embo J..

[39] Yaoxian W., Hui Y., Yunyan Z., Yanqin L., Xin G., Xiaoke W. (2013). Emodin induces apoptosis of human cervical cancer hela cells via intrinsic mitochondrial and extrinsic death receptor pathway. Cancer Cell Int..

[40] Di Marzo N., Chisci E., Giovannoni R. (2018). The role of hydrogen peroxide in redox-dependent signaling: Homeostatic and pathological responses in mammalian cells. Cells.

[41] Redza-Dutordoir M., Averill-Bates D.A. (2016). Activation of apoptosis signalling pathways by reactive oxygen species. Biochim. Biophys. Acta (Bba)-Mol. Cell Res..

[42] Liang Y., Lin Q., Huang P., Wang Y., Li J., Zhang L., Cao J. (2018). Rice bioactive peptide binding with TLR4 to overcome H_2_O_2_-induced injury in human umbilical vein endothelial cells through NF-κB signaling. J. Agric. Food Chem..

[43] Zhang Z., Jiang S., Zeng Y., He K., Luo Y., Yu F. (2020). Antioxidant peptides from Mytilus Coruscus on H_2_O_2_-induced human umbilical vein endothelial cell stress. Food Biosci..

